# Expression of BAG1 is associated with prognosis in kidney renal clear cell carcinoma based on bioinformatics

**DOI:** 10.1186/s12885-021-07874-w

**Published:** 2021-02-13

**Authors:** Hongrong Wu, Minjing Liu, Yuejun He, Guozhao Meng, Wanbei Guo, Qiong Guo

**Affiliations:** 1 Department of Pathology, the Affiliated Hospital of Guangdong Medical University, Zhanjiang, 524001 Guangdong China; 2 Institute of Basic Disease Sciences, XiangNan University, Chenzhou, Hunan Province China; 3 Department of Supervision, Baiyun International Airport Customs’ Inspection, Guangzhou, China; 4 Department of Urology, Hunan Provincial People’s Hospital, The First Affiliated Hospital of Hunan Normal University, No 61 West Liberation Road, Changsha, 410005 Hunan China

**Keywords:** BAG1, Prognosis, KIRC, Fatty acid metabolism, Oxidative phosphorylation

## Abstract

**Background:**

BCL2 associated Athano-Gene 1 (BAG1) has been described to be involved in the development and progression of cancer. But the role of BAG1 in kidney renal clear cell carcinoma (KIRC) has remained largely unknown.

**Methods:**

We performed bioinformatic analysis of data from TCGA and GEO dataset. The role of BAG1 in KIRC was explored by Logistic and Cox regression model. The molecular mechanisms of BAG1 was revealed by GSEA.

**Results:**

The current study found that the KIRC tumor samples have a low level of BAG1 mRNA expression compared to the matched normal tissues based on TCGA data and GEO databases. Low expression of BAG1 in KIRC was significantly associated with Sex, clinical pathological stage, tumor-node-metastasis (TNM) stage, hemoglobin levels, cancer status and history of neoadjuvant treatment. Kaplan-Meier survival analysis indicated that KIRC patients with BAG1 high expression have a longer survival time than those with BAG1 low expression (*p* < 0.000). Cox regression analysis showed that BAG1 remained independently associated with overall survival, with a hazard ratio (HR) of 1.75(CI:1.05–2.90; *p* = 0.029). GSEA indicated that the signaling pathways including fatty acid metabolism and oxidative phosphorylation were differentially enriched in high BAG1 expression phenotype.

**Conclusions:**

These findings suggested that BAG1 expression may act as a potential favorable prognostic marker and challenging therapeutic target.

## Background

Clear cell renal cell carcinoma (ccRCC) is the most common, lethal subtype of kidney cancer [1, 2]. There are five angiogenesis inhibitors approved by the United States Food and Drug Administration (FDA) for treatment of advanced and metastatic RCC, four of which VEGFR targeted tyrosine kinase inhibitors (TKIs) [2–4]. Despite the improved outcomes shown in the clinical trials of VEGF-targeted therapy, the length of response and survival benefit of therapy varies considerably among patients [3]. Therefore, it is urgent to explore the molecular target for patient prognosis and treatment.

BAG1 is a multifunctional protein which associates with cellular processes: apoptosis, proliferation, cell survival, and motility [5]. In recent years, increasing evidence has indicated that the pivotal role of BAG1 involved in the pathogenesis or progression of tumor [6–9]. Over-expression of BAG1 in head and neck squamous cell carcinomas (HNSCC) is associated with cisplatin-resistance [10]. BAG1 act as a potential prognostic marker in node-negative breast carcinoma [11]. However, to our best knowledge, the role of BAG1 in KIRC has not been described for the present.

In this study, we illustrate the prognostic value of BAG1 expression in KIRC using The Cancer Genome Atlas (TCGA) and gene expression omnibus (GEO) databases, the result shows that BAG1 expression associated with a well prognosis in patients with KIRC. At the same time, we conducted Gene Set Enrichment Analysis (GSEA) tool to identify the BAG1-related enriched signaling pathways in KIRC.

## Methods

### TCGA and GEO data

The gene expression (Workflow Type: level 3 HTSeq-FPKM) of 611 samples including 539 tumor samples, 72 normal samples and clinical data were collected from The Cancer Genome Atlas (TCGA). Boxplots method was used to test the discrete variables in KIRC tumor samples [12]. Finally, The RNA-Seq expression data of 449 patients with KIRC and clinical data were retained for further analysis (Table 1).

**Table 1:** Kidney renal clear cell carcinoma patient characteristics from TCGA data

Clinical characteristics		Total (449)	%
Age at diagnosis(y)		60 (26–90)	
Sex	male	289	64.4
	female	160	35.6
Grade	G1	12	2.7
	G2	189	42.4
	G3	173	38.8
	G4	70	15.7
	Gx	2	0.4
	NA	3	
Cancer status	With tumor	126	29.5
	Tumor free	296	69.3
	Discrepancy	5	1.2
	NA	22	.
Stage	I	219	49.1
	II	45	10.1
	III	106	23.8
	IV	76	17
	NA	3	
Tumor size	T1	225	50.1
	T2	56	12.5
	T3	159	35.4
	T4	9	2
Lymph nodes	Negative	199	93.4
	Positive	14	6.6
	NA	236	
Distant metastasis	Negative	366	83.2
	Positive	74	16.8
	NA	9	
Hemoglobin result	Elevated	4	1
	Low	227	59
	Normal	154	40
	NA	64	
History of neoadjuvant treatment	No	435	96.9
	Yes	14	3.1
Serum calcium result	Elevated	8	2.6
	Low	176	56.2
	Normal	129	41.2
	NA	136	

We also downloaded another gene expression data of renal tumors from the GEO database (the accession number: GSE105288) to provide further evidence for comparing gene expression between normal and tumor groups in KIRC patients.

### Gene expression analysis

BAG1 mRNA expression levels were extracted from the TCGA and GEO database using tidyverse package that is an opinionated collection of R packages designed for data science. Secondly, we employed ggplot2 and boxplot R packages to visualize expression levels of a single gene in different situation.

### GEPIA (Gene Expression Profiling Interactive Analysis)

GEPIA: a web server for cancer and normal gene expression profiling and interactive analyses [13].GEPIA performs overall survival (OS) or disease-free survival (DFS, also called relapse-free survival and RFS) analysis based on gene expression [13]. Thus, we employ online GEPIA tool to analyze the survival of KIRC patients.

### Gene Set Enrichment Analysis (GSEA)

GSEA is a computational method that determines whether an a priori defined set of genes shows statistically significant, concordant differences between two biological states [14]. This method using the GSEA tool was the same as those described by Wuhongr et al. [15]. In the present work, All the tumor samples were ranked according to the expression level of BAG1. The expression level was divided into high- and low- groups according to the median value of BAG1. Gene set permutations was set at 1000 times. The nominal *P* value and normalized enrichment score (NES) were used to sort the pathways enriched in each phenotype.

### Statistical analysis

All statistical analyses were performed out using the software R (3.5.1). The relationship between BAG1 as a continuous variable and clinical characteristics were explored by the Wilcoxon signed-rank test or the Kruskal-Wallis test. Association of BAG1 as a categorical variable with the clinicopathological characteristics of KIRC was explored through logistic regression. The cutoff value of BAG1 expression was obtained by the median expression level. Based on the cutoff value, the clinical tumor samples were stratified into “low” and “high” groups. Comparison of Kaplan-Meier survival between groups was performed using the log-rank statistics. Both univariate and multivariate analyses were performed to evaluate the prognostic significance of BAG1 expression in KIRC tumor patients.

## Results

### KIRC patients’ characteristics

The clinical information and BAG1 expression data of 449 KIRC patients from TCGA were merged into a document. Table 1 indicated that the median age at diagnosis is 60 years old. Female patients accounted for 35.6% (*n* = 160), male in 64.4% (*n* = 289). The clinical stage I was found in 219 patients (49.1%), stage II in 45 (10.1%), stage III in 106 (23.8%), stage IV in 76 (17%). 14 out of 213 (6.6%) cases had lymph node metastases. Distant metastases in 74 accounted for 16.8%. The median follow-up duration for subjects alive at last contact was 38.6 months (range 0–149 months).

We also collected gene expression data from renal tumors and normal kidneys in GEO data (series GSE105288) to detect the difference in BAG1 expression levels between normal and tumor group. The GSE105288 data include 9 normal samples,10 primary renal cell carcinoma samples and 35 metastasis renal cell carcinoma samples.

### Downregulation of BAG1 in KIRC tissues

**Fig. 1:**
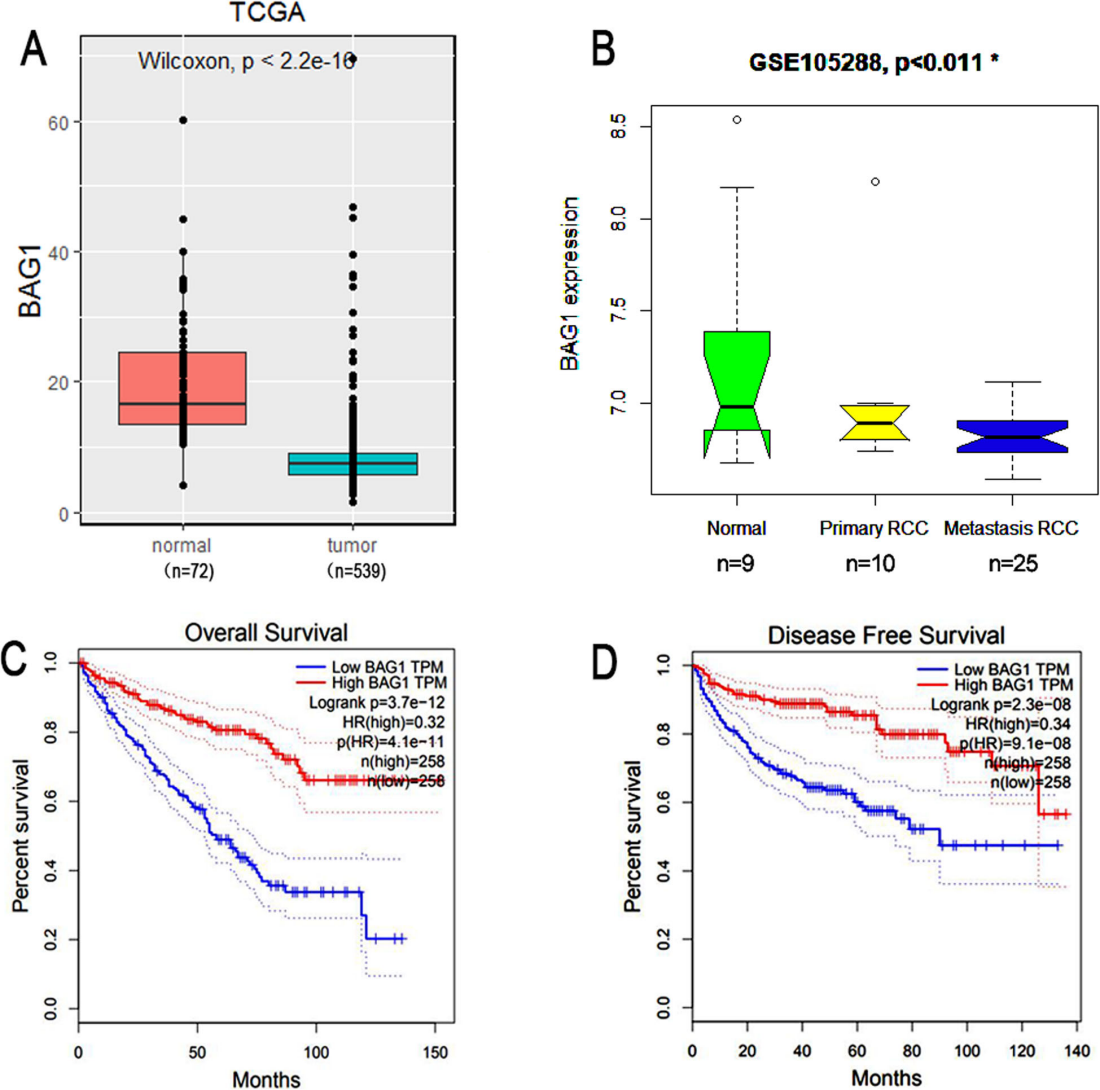
Downregulation of BAG1 expression in KIRC and survival analysis. **a** BAG1 mRNA expression in TCGA KIRC tissues and adjacent normal tissues. **b** BAG1 expression in GEO Dataset. **c**-**d** BAG1 correlated with OS and DFS in TCGA KIRC patients (*P* < 0.001)

KIRC tissues (*N* = 539) exhibited lower levels of BAG1 mRNA expression (*p* < 0.001) than the corresponding normal tissues (*N* = 72) in TCGA data (Fig. 1a). To further strengthen the evidence differences in BAG1 expression level between tumor and normal tissues, we retrieved another gene expression data from GEO (series number: GSE105288). Figure 1b showed that the BAG1 expression was also reduced in the tumor tissues of KIRC (*N* = 35, *p* < 0.011) compared with the normal tissues (*N* = 12).

### Association with BAG1 expression and pathologic variables

**Fig. 2:**
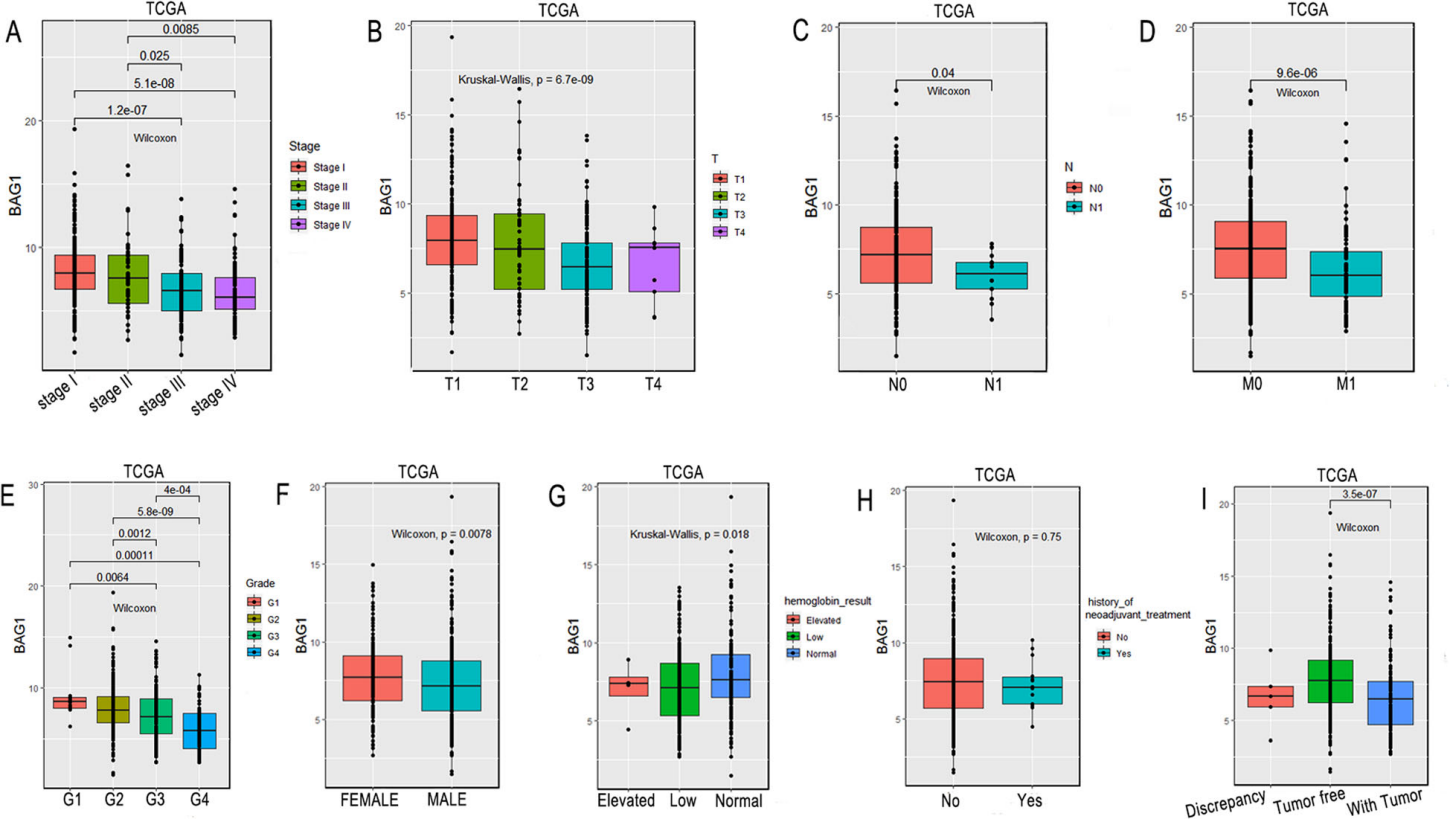
Association between BAG1 expression and clinicopathologic variables of patients with KIRC in TCGA. **a**-**i** BAG1 mRNA expression as a continuous variable significantly correlated with clinical stage, tumor size, lymph node metastasis, distant metastasis, grade, gender, serum hemoglobin level and cancer status

A total of 499 KIRC samples with BAG1 expression data and patient’s clinical pathological parameters from TCGA were analyzed. Unlike some previous studies that have suggested BAG1 may be associated with a poor clinical features [16, 17], we found that its expression as a continuous variable showed a significant inverse correlation with the clinical characteristics in KIRC, such as stage (stage I vs. stage IV *p* < 0.001), tumor size (*p* < 0.001), Sex (*p* < 0.001), grade (*p* < 0.001), serum hemoglobin level (*p* = 0.018), cancer status (*p* < 0.01), lymph node metastasis (*p* = 0.04) and distant metastasis (*p* < 0.001) (Fig. 2a-i). To further characterize this association between BAG1 and this clinical pathologic feature, We used logistic regression to explore the association of BAG1 expression as a categorical variable based on the median value of gene expression level with the clinicopathological characteristics of KIRC. Table 2 showed that there was also a significant correlation between BAG1 and tumor size (*p* < 0.001), lymph node metastasis (*p* < 0.01), distant metastasis (*p* < 0.001), clinical stage (*p* < 0.000), grade (*p* < 0.000), Sex (*p* < 0.01), hemoglobin levels (*p* < 0.01) and cancer status (*p* < 0.001).

**Table 2:** Association of BAG1^a^ expression with the clinicopathological characteristics of KIRC using logistic regression

Feature	OR (CI)	*p* value
Tumor size	1.84 (1.50 ~ 2.26)	0.000***
Lymph node metastasis	4.76 (1.04 ~ 21.81)	0.044*
Distant metastasis	3.71 (2.10 ~ 6.55)	0.000***
Clinical stage	1.72 (1.45 ~ 2.04)	3.64e-10***
Grade	2.18 (1.67 ~ 2.85)	0.000***
Age (continuous variant)		0.524
Gender (Male vs. Female)	1.88 (1.27 ~ 2.79)	0.001**
Hemoglobin result	0.57 (0.39 ~ 0.85)	0.006**
History of neoadjuvant treatment (Yes vs. No)		0.594
Cancer status	2.73 (1.76 ~ 4.23)	0.000***
Serum calcium (Low vs. High)		0.186

^a^Categorical dependent variable, greater or less than the median expression level

*:*p* < 0.05,**:*p* < 0.01,***:*p* < 0.001

### Survival outcomes and multivariate analysis

As shown in Fig. 1c and d, KIRC patients with higher BAG1 expression experienced significantly favorable overall survival (OS) (*p* < 0.001) and disease-free survival (DFS) (*p* < 0.001) than those with lower BAG1 expression through GEPIA tool. Table 3a showed that the univariate analysis using Cox regression indicated that high BAG1 expression correlated significantly with a good OS (hazard ratio [HR]: 2.11; 95% confidence interval [CI]:1.5–2.97; *p* < 0.001). Other variables associated with overall survival include Sex, clinical grade, stage, cancer status, and tumor size. At multivariate analysis, BAG1 remained independently associated with overall survival, with a HR of 1.75(CI:1.05–2.90, *p* = 0.029), along with tumor size, stage, lymph nodes and distant metastasis. Together, the results indicate that elevated BAG1 expression is a good prognosis for KIRC patients.

**Table 3:** Associations with overall survival and clinicopathologic characteristics in TCGA patients using Cox regression. b. Multivariate survival model after variable selection

Clinicopathologic variable	HR (95% CI)	*p*-value
a		
Age (continuous)	1.03 (1.02–1.05)	0.000 ***
BAG1 expression (Low vs. High)	2.11 (1.5–2.97)	0.000 ***
Grade	2.25 (1.83–2.77)	0.000 ***
Stage	1.94 (1.72–2.22)	0.000 ***
Cancer Status (tumor free vs. with tumor)	4.92 (3.52–6.91)	0.000 ***
Tumor size	1.90 (1.61–2.31)	0.000 ***
Lymph nodes (positive vs. negative)	3.41 (1.69–6.90)	0.000 ***
Distant metastasis (positive vs. negative)	4.43 (3.18–6.61)	0.000 ***
Gender (male vs. Female)	0.91 (0.65–1.31)	0.576
hemoglobin result (Low vs. Normal)	0.45 (0.30–0.66)	0.000 ***
history of neoadjuvant treatment (Yes vs. No)	2.37 (1.21–4.66)	0.012 *
Serum calcium (Low vs. High)	1.21 (0.81–1.61)	0.431
b		
Tumor size	1.25 (0.72–2.17)	0.417
Lymph nodes (positive vs. negative)	1.71 (0.78–3.73)	0.175
Distant metastasis (positive vs. negative)	1.97 (0.79–4.91)	0.145
Stage	1.25 (0.67–2.32)	0.478
BAG1 expression (Low vs. High)	1.75 (1.05–2.90)	0.029*

*:*p* < 0.05,**:*p* < 0.01,***:*p* < 0.001

### The BAG1-associated pathways analyzed by GSEA

**Fig. 3:**
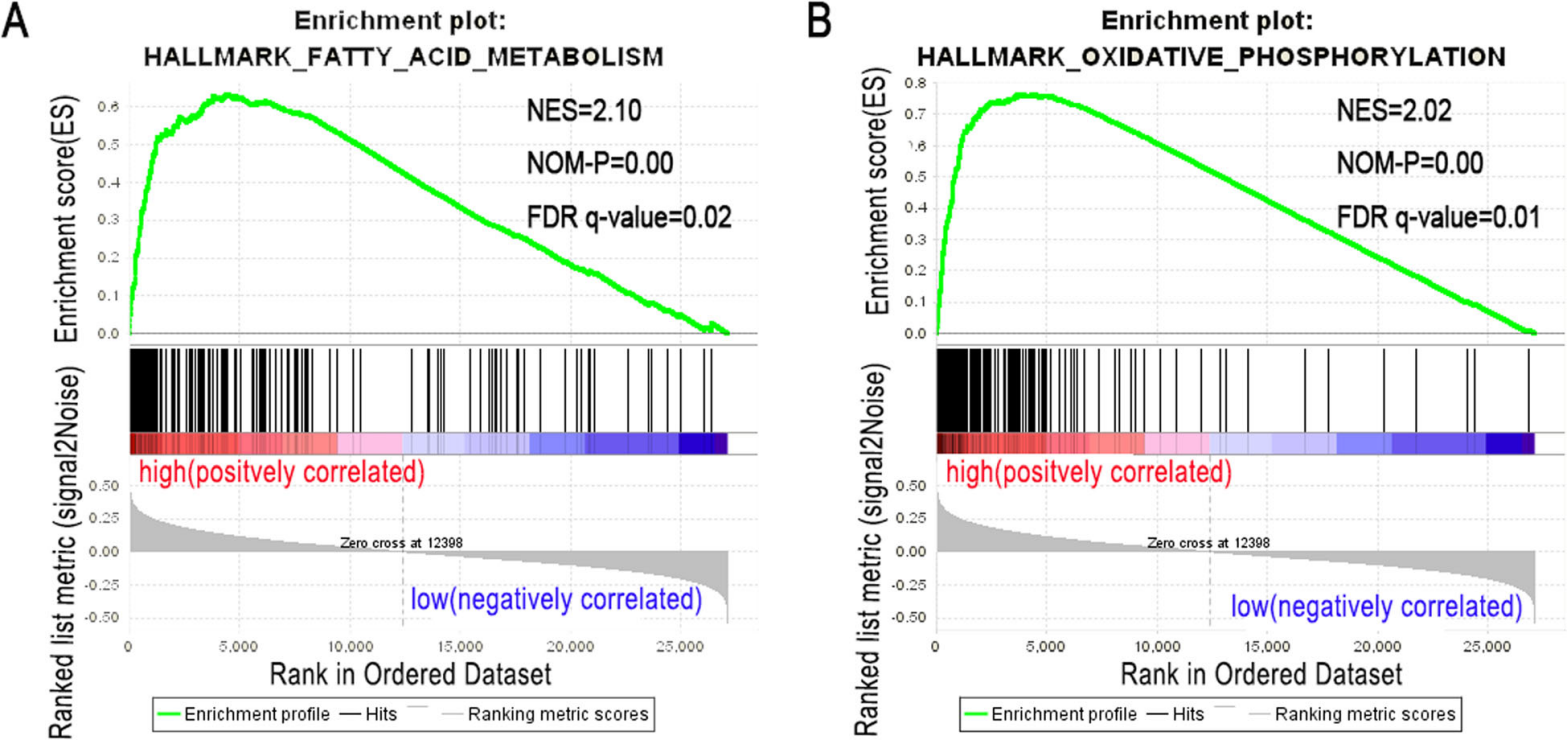
Enrichment plots from gene set enrichment analysis (GSEA). GSEA results showing some pathways such as fatty acid metabolism and oxidative phosphorylation are differentially enriched in BAG1-related KIRC. The enrichment score (ES, green line) means the degree to which the gene set is overrepresented at the top or bottom of the ranked list of genes

To search the biological mechanism of BAG1 in KIRC, we performed the GSEA to identify the tumor-associated pathways or gene sets that altered between the patients with low BAG1 expression and those with high BAG1 expression. We selected the most significantly enriched signaling pathways based on those principles such as their normalized enrichment score (NES), nominal *p* value< 0.05 and FDR < 0.25. Figure 3 showed that two signaling pathways (fatty acid metabolism and oxidative phosphorylation) are differentially enriched in high BAG1 expression group.

## Discussion

In the study, we demonstrated that the BAG1 mRNA expression levels were downregulated in KIRC patients compared to the normal through TCGA and GEO data (Fig. 1a, b). This result suggests that BAG1 may act as an inducible tumor suppressor in KIRC. However, our finding is interestingly inconsistent with other previous studies concerning the biological function of BAG1 [6, 10, 18–20].

In general, The oncogene BCL2 is a membrane protein that blocks a step in a pathway leading to apoptosis or programmed cell death [21]. BAG1 enhances the anti-apoptotic effects of BCL2 and act as a link between growth factor receptors and anti-apoptotic mechanisms [22]. Overexpression of BAG-1 has closely related to cell differentiation and TNM stage in esophageal cancer and its downregulation inhibits the proliferation and invasion of human esophageal carcinoma cells [16]. Thus, the BAG1 was considered to be a most commonly activated oncogene. However, inconsistent with other studies, our observations showed that low BAG1 expression in KIRC correlated significantly with aggressive tumor progression toward more malignant phenotypes including clinical stage, grade, tumor size, lymph node metastasis and distant metastasis (Table 2 and Fig. 2). these findings strongly suggested that BAG1 was a tumor suppressor gene and an attractive therapeutic target to inhibit cancer progression in KIRC. Therefore, these studies suggest that the BAG1 gene may have a dual role in tumorigenesis and need further study to support our findings.

In this study, KIRC patients with higher BAG1 expression have a significantly better overall survival (OS) and disease-free survival (DFS) than those with lower BAG1 expression (Fig. 1c, d). The univariate analysis and multivariate analysis using Cox regression show that BAG1 expression is a good prognosis for KIRC patients. This result indicated that BAG1 may be a prognostic marker and promising therapeutic target for KIRC patients. In addition, the present study also indicated that BAG1 has a dual role in tumorigenesis to some degree.

We found that the dual role of BAG1 may be due to its subcellular localization by searching associated references. For example, positive cytoplasmic BAG1 had a trend for favorable outcome following radical resection of pancreatic head cancer [23]. Nuclear BAG1 was highly expressed in HCC, and this overexpression was correlated with the poor prognosis as well as histological grade, suggesting a prognostic value of BAG1 in HCC [24]. Thus, we infer that BAG1 may inhibit cancer progression in KIRC vial localizing in the cytoplasm.

To explore the molecular mechanisms of BAG1 in KIRC, we conducted the GSEA tool to dig data mining for KIRC based on gene expression datasets from TCGA.GSEA identified that two signaling pathways (fatty acid metabolism and oxidative phosphorylation) are differentially enriched (Fig. 3). The general consensus is that these pathways take place in the mitochondria membrane [25, 26]. We therefore assumed that the subcellular location of BAG1 playing a role in KIRC is also located in the mitochondria membrane.

The present study has some limitations due to its pure bioinformatic method. The BAG1 expression level may not well represent its encoding protein level. Therefore, further experiments are required to assess the roles of BAG1 in KIRC.

## Conclusion

This study indicated that BAG1 expression is a favorable prognosis for KIRC patient and provides insight into the treatment of KIRC that BAG1 may serve as a potential therapeutic target in regulating several signaling pathways.

## Data Availability

Data collections and processions were performed according to policies of TCGA project (https://portal.gdc.cancer.gov/) and GEO (accession number: GSE105288). The public access to the databases is open.

## Abbreviations

BAG1: BCL2 associated Athano-Gene 1
KIRC: Kidney renal clear cell carcinoma
TCGA: The Cancer Genome Atlas
GEO: Gene expression omnibus
GSEA: Gene Set Enrichment Analysis
TNM: Tumor node metastasis
ccRCC: Clear cell renal cell carcinoma
FDA: Food and Drug Administration
TKIs: Tyrosine kinase inhibitors
HNSCC: Head and neck squamous cell carcinomas
OS: Overall survival
DFS: Disease-free survival
GEPIA: Gene Expression Profiling Interactive Analysis
